# Cassava Endophytic Bacteriome as Potential Biocontrol Agents Against Three Crop Phytopathogenic Fungi

**DOI:** 10.1002/mbo3.70254

**Published:** 2026-02-23

**Authors:** Roselyne Nyawir Owino, Edward K. Nguu, George O. Obiero, Evans N. Nyaboga

**Affiliations:** ^1^ Department of Biochemistry University of Nairobi Nairobi Kenya; ^2^ Centre for Biotechnology and Bioinformatics University of Nairobi Nairobi Kenya

**Keywords:** bacterial endophytes, biocontrol agents, cassava varieties, crop fungal diseases, GC‐MS analysis

## Abstract

Crop yield losses stemming from pathogen infection and pests constitute 10%−40% of the potential annual total world crop production. Biological control agents have gained prominence as an environmentally friendly alternative to the use of hazardous synthetic agrochemicals. Herein, we evaluated the potential role of endophytic bacteria from cassava as biological control agents of three crop phytopathogenic fungi. Eighty‐four endophytic bacteria isolated from cassava were assessed for antagonistic activity against phytopathogens *Colletotrichum siamense*, *Colletotrichum sublineola*, and *Phytophthora infestans* infecting cassava, sorghum and potato, respectively. Fourteen endophytic bacteria exhibited antifungal activity against the three phytopathogens. Of the 14 endophytic bacteria, four [*Bacillus siamensis* AS3, *B. velezensis* (DP1 and CS3b) and *B. subtilis* DL6] demonstrated significantly high inhibition rate on mycelial growth ranging from 62% to 72.3% against *C. siamense*, 63%–65.9% against *C. sublineola* and 64%–75.3% against *P. infestans* as compared with the control. The four endophytic bacteria produced volatile organic compounds that inhibited fungal growth ranging from 34.1% to 46.4% in *P. infestans*, 31.9%–36% in *C. siamense* and 33.9%–39.6% in *C. sublineola*. The results highlight the biocontrol potential of endophytic bacteria from cassava against three crop phytopathogenic fungi, which could be used for the future development of sustainable management strategies using environmentally friendly approaches. This study demonstrated that *B. siamensis*, *B. velezensis*, and *B. subtilis* with a strong antagonistic effect on *C*. *siamense*, *C*. *sublineola*, and *P. infestans*, can be potential biocontrol agents for cassava and sorghum anthracnose and potato late blight.

## Graphical abstract

Eighty‐four endophytic bacteria from cassava were assessed for antagonistic activity against phytopathogens *Colletotrichum siamense*, *Colletotrichum sublineola* and *Phytophthora infestans* infecting cassava, sorghum and potato, respectively. Fourteen endophytic bacteria exhibited antifungal activity against the three phytopathogens, of which four demonstrated the significantly high mycelial growth inhibition as compared with the control.

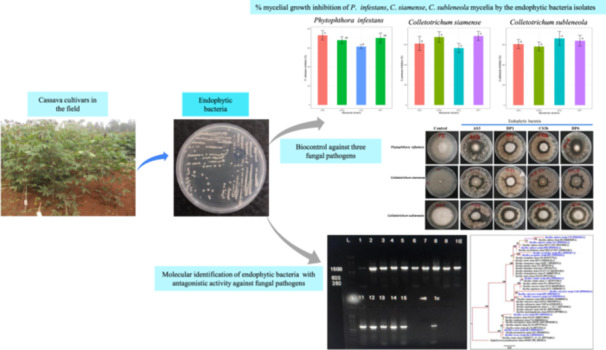

## 1 Introduction

Plant pathogens and pests cause global yield losses of up to 40% of staple crops and result in production losses of hundreds of billions of dollars (Rizzo et al. 2021). Diseases caused by fungal pathogens contribute approximately 20%−40% of these losses causing production losses of $100−200 billion annually in staple crops including cassava, sorghum and potato (Li et al. 2024). Crop fungal pathogens causing great yield losses include *Colletotrichum* spp., *Fusarium* spp., and *Phytophthora* spp. (Doehlemann et al. 2017).

The genus *Colletotrichum* is one of the most significant fungal pathogens affecting a wide range of staple and economical important crops in Africa. One of the most destructive fungal diseases in cassava, cassava anthracnose disease (CAD) is caused by several *Colletotrichum* spp. including *C. siamense*, *C. fruticola* and *C. gloeosporioides* (Ng'ang'a et al. 2019; William et al. 2012). The disease is characterized by stem, branch, and fruit cankers, leaf spots, and tip die‐backs on aerial plant parts (Obilo et al. 2010). The disease results to low germination rates of 40%−60% from severely infected stems, while infected seed materials result in loss of seed viability of 50%−75%. The disease reduces the amount of healthy planting materials and can cause total yield loss as reported from Democratic Republic of Congo and Thailand (Sangpueak et al. 2021). Cassava brown leaf spot disease (BLSD), also caused by *Colletotrichum* species has been reported to cause root yield losses of 30% in Africa, 17% in India and 23% in South America (Powbunthorn et al. 2012). Other important crop fungal pathogens include sorghum anthracnose pathogen *Colletotrichum sublineola* and potato late blight *Phytophthora infestans*. Sorghum anthracnose caused by *C. sublineola* is the most damaging foliar fungal disease of sorghum and can lead to 100% yield loss (Koima et al. 2023). Potato late blight disease caused by the fungal pathogen, *P. infestans* is the most devastating disease and key biotic factor affecting potato production globally (Dong and Zhou 2022). *Phytophthora infestans* causes significant annual crop losses of approximately $10 billion and is one of the most aggressive plant pathogens (Ivanov et al. 2021; Haverkort et al. 2009).

Chemical methods have for many years been used in the control of fungal pathogens, however, their use presents heavy economic costs and environmental pollution. Their excessive use has also resulted in harmful effects including the emergence of resistance by pathogens, chemical residues in the soil, effects on non‐target plants, animals and other soil microbes (such as nematodes, non‐pathogenic fungi and bacteria), and a risk to human health (Goswami et al. 2018). As these chemical strategies become less effective with undesirable effects, there is a need for more ecologically sustainable pathogen control measures. Currently, one of the main objectives of the African Union is to reduce the chemicals used in agriculture in order to minimize their negative impact on the environment, and to consequently produce safer agricultural products for human consumption. Consequently, green and safe biological control methods such as the utilization of antagonistic endophytic bacteria have garnered increased attention by the scientific community. Endophytic bacteria have attracted interest as biological control agents (BCAs) because they not only inhibit the growth of pathogens and induce plant disease resistance but also align with the public demand for food safety and environmental friendliness (Das et al. 2025; Baard et al. 2023; Pérez‐Montaño et al. 2014).

Endophytic bacteria offer great untapped potential as BCAs due to their ability to colonize same ecological niche as phytopathogens (Glick 2014; Pérez‐Montaño et al. 2014). Among these, *Bacillus* species are commonly used as a reservoir of biocontrol agents to manage a variety of plant diseases. They demonstrate great antagonistic potential by production of broad‐spectrum antimicrobial compounds such as extracellular lipopeptides, lytic enzymes, polyketides and volatile organic compounds (VOC), induction of systemic resistance in plants, inhibiting pathogen invasion, interfering with pathogen quorum sensing (QS) and competing with the pathogens for nutrients and/or niche (Card et al. 2016; Ajuna et al. 2024). *Bacillus siamensis*, *B. velezensis*, and *B. subtilis* isolated from a wide range of sources have been reported as plant pathogen control agents. *Bacillus velezensis* is a potential biological control agent against plant diseases, which is extensively found in nature including in plant tissues and distinguished by its capacity to adapt to diverse environmental conditions (Wockenfuss et al. 2024). Recent studies have positioned endophytic bacteria, *B. velezensis* as a promising candidate for biological control applications due its antagonistic activity against fungal phytopathogens (Kenfaoui et al. 2024; Tu et al. 2024). The endophytic bacterium synthesizes antimicrobial compounds such as hydrolases, bacteriocins, lipopetides (e.g., iturin A, surfactin), VOCs and polyketides that can suppress plant pathogens (Kenfaoui et al. 2024; Fan et al. 2018).

*Bacillus subtilis* is particularly notable due to its various modes of action and environmental compatibility. It deploys a range of antagonistic mechanisms including the production of antifungal cyclic lipopeptides such as fencycin, surfactin and iturin A, which are effective against a broad range of crop pathogens. In addition, *B. subtilis* secretes a variety of enzymes such as proteases, β−1,3‐glucanase and cellulase that degrade fungal cell walls, as well as production of siderophores for sequestration of iron, a critical mineral nutrient for fungal growth (Saberi Riseh and Gholizadeh Vazvani 2024). *Bacillus subitlis* has also been reported to induce systemic resistance in plants as an indirect antimicrobial effect, triggering their defense mechanisms and enhance resistance to pathogen attacks (Saberi Riseh and Gholizadeh Vazvani 2024). *Bacillus siamensis* has also emerged as a promising biocontrol agent due its ability to promote plant growth and resistance through antibiosis and competitive exclusion (Sharma et al. 2021). For instance, *B. siamensis* isolated from various crops have been shown to have inhibitory properties on growth of various fungal pathogens of crops such as potato, rice and chickpea (Yang et al. 2023; Sharma et al. 2021; Gorai et al. 2021). Although previous studies have highlighted the potential of *B. siamensis*, *B. velezensis*, and *B. subtilis* to control fungal phytopathogens, the effects have been restricted to a few crops such as potato, rice, cotton, strawberry, lettuce, wheat and tomato (Yang et al. 2023; Sharma et al. 2021; Gorai et al. 2021; Fan et al. 2018).

The novelty of the present work lies in the detailed study of the endophytic bacteria from cassava for antifungal protection against *C. siamense*, *C. sublineola*, and *P. infestans*, an area not extensively explored. There is limited information on endophytic bacteria in cassava and their biocontrol potential against the three crop fungal phytopathogens. The study fills a critical gap in the literature, especially regarding the potential of endophytic *Bacillus* species from cassava to control *C. siamense*, *C. sublineola*, and *P. infestans* infecting cassava, sorghum and potato, respectively. The aim of the present study therefore was to isolate and characterize endophytic bacteria from different cassava cultivars and assess their potential role in the biocontrol of fungal pathogens causing diseases of economic importance in cassava, sorghum, and potato using *in vitro* antagonistic assays. Our results demonstrate the potential of endophytic bacteria from cassava in the biocontrol of three crop fungal phytopathogens.

## 2 Materials and Methods

### 2.1 Sample Collection

Healthy leaf, stem, and petiole samples were collected from 8‐month‐old plants of four cassava cultivars (MM08/2206, Samgoja, Karembo, and MM96/4884) grown in experimental plots at Kabete field station, University of Nairobi (1°14′51.19188”S 36°44′30.05412”E). The selected cultivars are resistant to anthracnose and leaf spot diseases infecting cassava (Ager 2023). During the growing season, the average of precipitation was 1200 mm and the temperature varied between 20°C and 30°C during the growing season. Fertilizers and pesticides were not applied during the experiment. The samples were cut from the plants using sterile blades, placed in sterile polypropylene bags and transported in an ice‐cool box to the Department of Biochemistry, University of Nairobi. Endophytic bacteria were isolated from the plant tissues within 2 h of sample collection.

### 2.2 Isolation of Cassava Endophytic Bacteria

The isolation of endophytic bacteria was carried out as described by Costa et al. (2012) with some modifications. Leaf, stem, and petiole samples were washed under running tap water for 2 min to remove surface debris, then surface sterilized using 70% ethanol for 1 min followed by disinfection using 2% sodium hypochlorite solution for 1 min. Samples were then rinsed three times with sterile distilled water and dried using sterile filter papers. To assess the effectiveness of the sterilization process, 1 mL of the last rinsing water was plated on nutrient agar (NA) media (HiMedia Laboratories, India). Plant samples were cut into small pieces of approximately 5 × 5 mm using sterile surgical blades and plated on nutrient agar (NA) media then incubated at 28°C for 2−3 days. The resulting morphologically different colonies of endophytic bacteria were selected and sub‐cultured on NA media plates to obtain pure colonies and then transferred to nutrient broth (NB) media (HiMedia Laboratories, India).

### 2.3 *In Vitro* Preliminary Screening of Endophytic Bacteria for Antifungal Activity

The endophytic bacteria isolates were screened for their ability to inhibit growth of cassava phytopathogenic fungi *Colletotrichum siamense* using the dual culture assay described by Sharma et al. (2021) with some modifications. *Colletotrichum siamense*, previously isolated from cassava brown leaf spots in a different study at the University of Nairobi (Ager 2023), was retrieved from glycerol stock. Fungal sample was inoculated at the centre of a potato dextrose agar (PDA) (HiMedia Laboratories, India) plate and incubated at 25°C for 7 days. The endophytic bacteria isolates were cultured in nutrient broth at 28°C for 48 h before use.

For the dual culture assay, a 6 mm diameter plug from the 7‐day‐old fungal culture was inoculated at the center of a PDA plate while 20 µL of pure endophytic bacteria suspension was inoculated around the fungal plug in a circular pattern, 1 cm from the edge of the plate. This was done individually for all the isolated endophytic bacteria, inoculating one pure bacterial isolate per plate. PDA plate inoculated with only the fungal plug was used as a control for the experiment. The plates were incubated at 28°C for 7 days. Diameter measurements of the fungal isolate in the control plates and in the treatment plates with endophytic bacteria were recorded. Endophytic bacterial isolates that showed inhibition of fungal growth were selected and the dual culture assay repeated with three biological replicates. The percentage growth inhibition of *Colletotrichum siamense* was calculated using the formula described by Sharma et al. (2021):

(1) $$\mathrm{GI}=\frac{A-B}{A}\times 100,$$

where GI = fungal growth inhibition;

*A* = Fungal diameter in control plate;

*B* = Fungal diameter in the presence of endophytic bacteria.

Bacterial strains with the highest antifungal activity (more than 30% fungal growth inhibition) were selected for molecular identification.

### 2.4 Molecular Identification of Antagonistic Bacterial Endophytes and Phylogenetic Analysis

The screened antagonistic bacterial endophytes were identified by 16S rRNA sequencing. The genomic DNA of antagonistic bacterial endophytes was first extracted using PureLink Genomic DNA Mini Kit (Thermo Fisher, Switzerland) according to the manufacturer′s instructions. DNA quality was assessed by running DNA samples on 1% agarose gel electrophoresis stained with ethidium bromide and visualized under a UV transilluminator.

The DNA samples were amplified by polymerase chain reaction (PCR) with bacterial 16S rDNA primers: forward B27F (5′‐AGAGTTTGATCCTGGCTCAG‐3′) and reverse 1492 R (5′‐TACCTTGTTACGACTT‐3′). PCR amplifications were carried out in a total volume of 25 µL containing 12.5 µL GoTaq Green Master Mix (Promega, USA), 0.5 µL each of forward and reverse primers, 10.5 µL of nuclease free water and 1 µL of DNA template (50 ng/µL). The PCR cycling conditions used were: initial denaturation at 95°C for 5 min followed by 35 cycles (denaturation at 95°C for 1 min, annealing at 57°C for 1 min and extension at 72°C for 1 min) and a final extension at 72°C for 7 min using a Bio‐Rad MJ Mini Gradient thermocycler 21 (catalogue #PTC‐1148). The amplified products were analyzed by 1% agarose gel electrophoresis. PCR products were sent to Macrogen, Netherlands for Sanger sequencing using both forward and reverse primers to obtain partial 16S rDNA sequences.

The sequence chromatograms obtained were transferred to the BioEdit Sequence Alignment Editor program version 7.2.5 for visualization and manual editing to obtain contiguous sequences (Hall 1999). The contig sequences were then subjected to Basic Local Alignment Tool (BLAST) searches in GenBank at NCBI for taxonomic identification and selection of sequences for phylogenetic analysis based on sequence identities with related bacteria in the database. Only sequences with over 90% sequence similarity with the query sequences and E values of 0.0 were chosen. Selected sequences alongside the query sequences were subjected to multiple sequence alignment using the Muscle program version 3.6. The Bayesian phylogenetic approach in MrBayes, version 3.1.2 (Huelsenbeck and Ronquist 2001) was used to perform phylogenetic analysis based on the nucleotide sequences. Phylogenetic inference was performed for 4,000,000 generations from which a tree was sampled every 1000 generations. The Bayesian consensus trees were constructed using the “sum p” and “sum t” options in MrBayes and the branching confidence was calculated as posterior probabilities. The trees were visualized in Figtree, version 1.4.3 obtained at http://tree.bio.ed.ac.uk/.

### 2.5 Screening Endophytic Bacteria for Antifungal Activity Against Three Crop Fungal Phytopathogens

The selected antagonistic endophytic bacteria against *C. siamense* were screened for antagonist activity against *C*. *sublineola* and *Phytophthora infestans* based on dual culture assay (direct), cell‐free supernatant (indirect) and VOCs (remote confrontation). The pathogens *C*. *sublineola* and *P. infestans* were isolated and characterized in previous studies (Koima et al. 2023; Mukri 2024) at the University of Nairobi.

#### 2.5.1 Dual Culture Plate Assays (Direct Confrontation)

Dual culture plate assays were performed as described by Sharma et al. (2021) with some modifications. The endophytic bacteria selected based on antagonistic screening against *C. siamense* were tested for their ability to inhibit growth of *Colletotrichum sublineola* and *Phytophthora infestans* infecting sorghum and potato, respectively. The direct confrontation assays were done as described in Section 2.3 and the percentage of fungal growth inhibition calculated using the formula in Section 2.3 (Equation 1) as described by Sharma et al. (2021).

#### 2.5.2 Antifungal Activity of Endophytic Bacteria Based on Bacterial Volatile Organic Compounds (Remote Confrontation)

The inverted petri dish microcosm set‐up previously described by Boiu‐Sicuia et al. (2023) was used to determine the antifungal activity of bacterial volatile organic compounds (VOCs). A 200 µL bacterial suspension was inoculated on a nutrient agar plate and spread evenly using sterile cotton inoculating swabs. The pathogenic fungi were prepared by inoculating a 6 mm plug of the 7‐day‐old fungi at the center of a PDA plate. The plate lids for both the endophytic bacterial and fungal plates were removed and the compartment inoculated with the pathogenic fungal isolate was placed in an inverted position over the compartment spread with the endophytic bacteria and then tightly sealed with parafilm. The control plates setup was inoculated with sterile nutrient broth in place of the bacteria. This was done for all the three fungal samples and the selected bacterial isolates. The microcosm set‐ups were incubated at 25°C for 7 days, after which fungal diameter measurements were taken. The antifungal efficacy of the bacterial VOCs was calculated as follows: Inhibition rate (%) = (colony diameter exposed to mock—colony diameter exposed to *Bacillus* VOCs)/colony diameter exposed to mock × 100%. Each treatment had five replications and the experiment repeated three times.

#### 2.5.3 Antifungal Activity of Endophytic Bacteria Based on Cell Free Supernatant (CFS) (Indirect Confrontation)

The preparation of CFS was done according to Liu et al. (2023) with slight modifications. Endophytic bacteria were cultured in nutrient broth for 72 h at 28°C then centrifuged at 8000 rpm for 20 min. The supernatant was collected and filtered through a 0.20 µm membrane filter to obtain the CFS in sterile tubes.

For each of the endophytic bacteria, 2 mL of the CFS solution (10% of culture media) was poured into each sterile petri plate followed by addition of 20 mL PDA at 40°C. For the control plates, PDA plates inoculated with 2 mL sterile nutrient broth were used. A 6 mm plug of each fungal isolate was inoculated at the center of each plate and incubated at 25°C for 7 days. Three replications were used for each treatment. Measurements of diameters of the pathogenic fungi were taken and antifungal efficacy of bacterial CFSs calculated using the formula in Equation 1 in Section 2.3 as described by Sharma et al. (2021).

### 2.6 Gas Chromatography‐Mass Spectrometry (GC‐MS) Analysis of Bacterial Volatile Compounds

Endophytic bacteria isolate AS3 was selected for GC‐MS analysis to identify bacterial volatile compounds. The isolate AS3 was cultured in nutrient broth for 24 h in an incubator‐shaker at 28°C before GC‐MS analysis was carried out. The sample (10 mL) was partitioned with 10 mL each of hexane, ethyl acetate, petroleum ether, benzene, butanol and methanol respectively. The solvent layers were pooled together and concentrated using high purity Nitrogen to 1 mL volume. 20 µL of the sample was subjected to GC‐MS analysis.

GC‐MS analysis was carried out using Agilent 7890 A Gas Chromatography coupled to an Agilent 5975 C mass spectrometer equipped with a DB5 capillary column, 30 m long, 0.25 µm wide and a 0.25 µm film thickness. The temperature of the column was programmed from 50°C (5 min) to 120°C, at a rate of 15°C/min, and the final temperature reached 250°C, at an increasing temperature rate of 10°C/min. Helium grade 5.0 was used as a carrier gas at a flow rate of 1 mL/min. Split/split less (split less mode) inlet temperature was 280°C as was that of the mass spectrometry transfer line. The temperature of the ion source was maintained at 230°C. The mass spectrometer was operated under electron ionization mode at 70 Ev. Mass spectra and the total on chromatograms were obtained by automatic scanning mass range (m/z) of 45,400. The volatile components were identified by comparing the obtained mass spectrum with data available in the NIST spectra library. The content of each compound was expressed as the relative area of the peak in the total ion chromatogram.

### 2.7 Effect of Temperature, Metal Ions, Salt, and Osmotic Stresses on Endophytic Bacteria

The ability of the selected endophytic bacteria to grow in different concentrations of salt and metal ions, osmotic stress (PEG), and temperatures was determined as described by Slama et al. (2019). For this purpose, the endophytic bacteria were grown in nutrient broth with different salt concentrations (0%, 1.5%, 3%, 4.5% and 6%) to determine their tolerance to salinity, polyethylene glycol (PEG) (0%, 2.5%, 5%, 7.5% and 10%) to determine their tolerance to osmotic stress, and CuSO_4_ (0, 50, 100, 150, 200 mg/L) to determine growth in the presence of metal (Cu^2+^) ions. Samples were incubated at 28°C for 48 h after which bacterial growth was determined by optical density measurements using a spectrophotometer at 600 nm. The ability of the bacterial isolates to grow at different temperature conditions was determined by inoculating the bacteria on NA media and incubation at the respective temperatures (10°C, 20°C, 30°C, 40°C and 50°C) for 48 h.

### 2.8 *In Vivo* Effects of Endophytic Bacteria on Cassava Growth

Endophytic bacteria were assayed for their effect on growth of cassava plants (KALRO Agric cultivar) under greenhouse conditions according to Ferreira et al. (2021) with modifications.

Stem cuttings of cassava were established in 9 cm diameter by 10 cm height plastic pots containing 200 g of forest soil. The sprouted plants were allowed to grow in the glasshouse conditions (temperature at 25°C–30°C with 60%–80% relative humidity) for 2 weeks before use in inoculation experiments.

Endophytic bacteria were cultured in nutrient broth for 24 h at 28°C before inoculation into cassava plants. The cultured bacteria then were centrifuged at 6000 rpm for 15 min and the supernatants were discarded. The bacterial pellet cells were suspended in saline solution (0.8% NaCl) followed by optical density measurements using a spectrophotometer at 600 nm. Optical density (OD) measurements were adjusted to OD 0.5 containing 2 × 10^9^ CFU/mL using saline solution, the resulting bacterial suspensions were used for inoculation of cassava plants.

The prepared endophytic bacterial suspensions were inoculated into 2‐week‐old cassava plants in the glasshouse. For each treatment, five plants were inoculated with drops of 2 mL of bacterial suspension and inoculation repeated after 14 days. The five control plants were provided with drops of 2 mL of the sterile saline solution and this repeated after 14 days. The 2 mL of bacterial suspension per plant was the volume required for complete and homogenous irrigation of 200 g of soil contained in each plastic pot. The inoculated and control plants were cultivated for 14 days under greenhouse conditions for further analysis. All plants were irrigated after every 2 days with water. Plant growth‐related parameters of cassava plants at 30 days post‐inoculation were measured and recorded.

### 2.9 Statistical Data Analysis

Percentages of inhibition were subjected to analysis of variance (ANOVA) using generalized linear models with the corresponding R packages in InfoStat v2008 (Houston, TX, USA). Normality and homoscedasticity were checked and corrected when necessary and means were separated using Fisher's least significant difference test (*p* < 0.05). Data were plotted in GraphPad Prism v.5.03 (San Diego, CA, USA).

## 3 Results

### 3.1 Isolation of Endophytic Bacteria From Cassava and Preliminary Screening of Antagonistic Activity Against *C. siamense*

Eighty four bacterial endophytes were isolated from leaf, stem and petiole tissues of the four cassava cultivars (MM08/2206, Samgoja, Karembo and MM96/4884) (Supporting Information S1: Table S1; Supporting Information S1: Figure S1) were tested in vitro against the growth of *C. siamense*. Fourteen endophytic bacteria isolates inhibited the growth of *C. siamense* at different degrees (mm) compared with the control (Table 1). The diameter of *C. siamense* in the presence of endophytic bacteria isolates ranged from 22 ± 0.08 to 35 ± 0.08 mm. The diameter of the control plates (*C. siamense* without the endophytic bacteria) was significantly higher (55 ± 0.08 mm, *p* < 0.05) compared with plates with both the pathogen (*C. siamense*) and endophytic bacteria (Table 1).

**Table 1:** The diameter of growth of *Colletotrichum siamense* in the presence of endophytic bacteria (antagonistic capability of the endophytic bacteria *in vitro*).

Cultivar	Tissue	Isolates of endophytic bacteria	*C. siamense* diameter (mm)^a^	Antagonistic effect
A (MM08/2206)	Leaf	AL3	27 ± 0.14c	++
	Stem	AS3	23 ± 0.19 d	+++
B (Samgoja)	Leaf	BL3	35 ± 0.1b	+
	Petiole	BP2	26 ± 0.08c	++
C (Karembo)	Leaf	CL1	25 ± 0.07 cd	++
	Petiole	CP3	28 ± 0.08c	++
	Stem	CS3b	22 ± 0.35 d	+++
	Stem	CS5	35 ± 0.04b	+
D (MM96/4884)	Leaf	DL3	27 ± 0.07c	++
		DL4	35 ± 0.08b	+
		DL6	23 ± 0.2 d	+++
	Petiole	DP1	22 ± 0.08 d	+++
		DP3	24 ± 0.07 cd	+++
		DP6	26 ± 0.07c	++
Control (*C. siamense* alone without endophytic bacteria)			55 ± 0.08a	

*Note:* “+++” means fungal diameter ˂ 25 mm, “++” means 25 mm > fungal diameter ˂ 30 mm, “+” means fungal diameter > 30 mm. Data presented are diameters of *C. siamense* cultured in the presence of endophytic bacteria. The control group was *C. siamense* cultured in the absence of the endophytic bacteria. Data represents mean of diameters ± SD (*n* = 3).

a Same lower case letter(s) in column indicates not significantly different according to Duncan's multiple range test (*p* = 0.05).

### 3.2 Molecular Identification of Antagonistic Endophytic Bacteria Using 16S rRna

The 14 antagonistic bacterial isolates were identified using ribosomal region 16S rRNA. The resulting sequences were compared in the NCBI database using the BLAST program. Sequence analysis of the 12 endophytic bacteria isolates revealed 97%–100% match to the genus *Bacillus*, which were designated as *B. siamensis*, *B. velezensis*, *B. subtilis*, *B. safensis*, *B. cereus*, *B. stratosphericus*, and *B. altudinis*. Sequence analysis of the two endophytic bacteria isolates revealed 99% and 100% match to *Staphylococcus saprophyticus* (Table 2).

**Table 2:** Cassava endophytic bacteria identification and parameters from BLASTn sequence analysis in comparison with known homologous sequences from NCBI database.

Isolate	GenBank Accession (This study)	Closest type strain	Query coverage (%)	E value	Percentage Identity	Sequence Length	GenBank Accession (Reference strain)
AS3	PP855614.1	*Bacillus siamensis*	100	0.0	97.43	1401	ON908919.1
DP1	PP855615.1	*Bacillus velezensis*	100	0.0	99.23	1520	KJ767354.1
CS3b	PP855616.1	*Bacillus velezensis*	99	0.0	97.60	1425	MN560069.1
DL6	PP855622.1	*Bacillus subtilis*	100	0.0	99.64	1419	KY072760.1
DP6	PP855623.1	*Bacillus safensis*	100	0.0	99.79	1454	MN704399.1
CL1	PP855624.1	*Bacillus safensis*	100	0.0	99.48	1417	JX183149.1
CS5	PP855625.1	*Bacillus safensis*	99	0.0	99.55	1461	OR056185.1
DL3	PP855619.1	*Bacillus cereus*	100	0.0	99.93	1512	PP754460.1
DP3	PP855621.1	*Bacillus cereus*	100	0.0	98.39	1482	MT510174.1
DL4	PP855620.1	*Bacillus cereus*	99	0.0	100	1512	PP754460.1
BL3	PP855617.1	*Bacillus stratosphericus*	99	0.0	97.52	1399	MH057391.1
BP2	PP855618.1	*Bacillus altitudinis*	100	0.0	98.00	1423	MG651148.1
CP3	PP855627.1	*Staphylococcus saprophyticus*	100	0.0	99.78	1400	MT539740.1
AL3	PP855626.1	*Staphylococcus saprophyticus*	99	0.0	100	1400	MT539740.1

**Figure 1:**
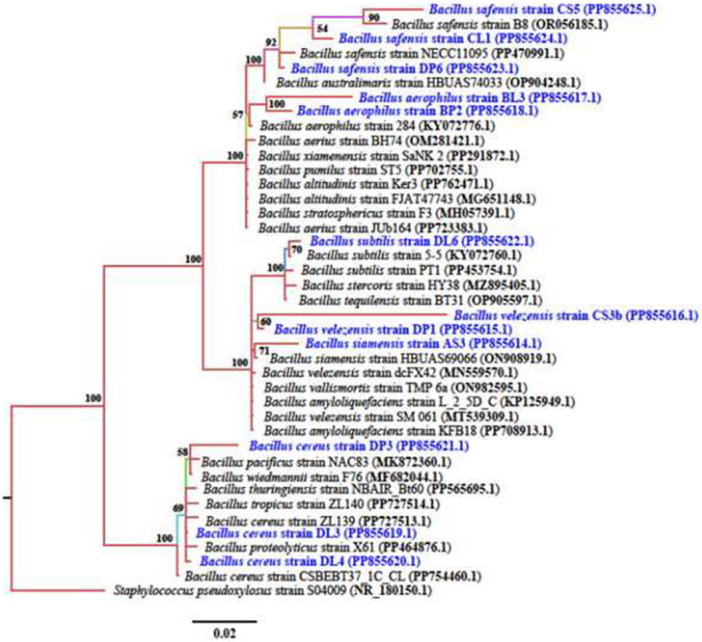
Phylogenetic tree showing evolutionary relationships of bacterial endophytes (*Bacillus* spp.). Tree building was done using the Markov chain Monte Carlo (MCMC) method using MrBayes software. Numbers at the nodes indicate the percentage of posterior probabilities indicating the topological robustness of the Phylogenetic tree. Isolates from this study are presented in blue. *Staphylococcus pseudoxylosus* strain S04009 was used as an outgroup to root the tree.

Phylogenetic tree construction showed close relation between the endophytic bacterial isolates from the current study and known sequences from NCBI database. The phylogenetic analysis showed that isolate AS3 was closest related with *B. siamensis* of accession number ON908919.1 while isolates CS3b and DP1 were closely related with *B. velezensis* of accession number MN559570.1. Isolate DL6 was closely related with *B. subtilis* KY072760.1. Isolate CL1 clustered with isolate CS5 and *B. safensis* of accession number PP855625.1. while isolate DP6 formed a monophyletic clade with *B. safensis* of accession number OP904248.1. Isolate BL3 and BP2 formed a monophyletic clade with each other and clustered with *B. aerophilus* of accession number KY072776.1. Isolates DP3, DL3, and DL4 clustered with *B. cereus* of accession number PP727514.1 (Figure 1).

**Figure 2:**
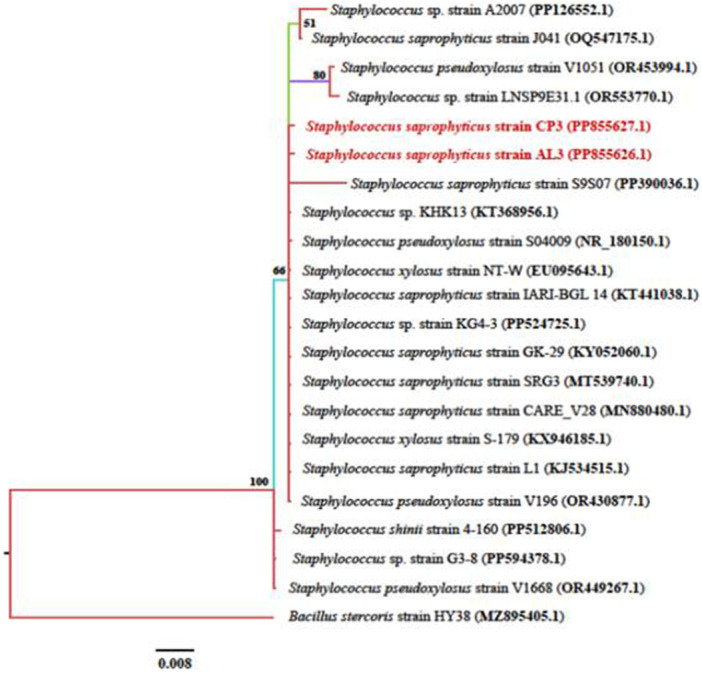
Phylogenetic tree showing evolutionary relationships of bacterial endophytes (*Staphylococcus spp.*). Tree building was done using the Markov chain Monte Carlo (MCMC) method using MrBayes software. Numbers at the nodes indicate the percentage of posterior probabilities indicating the topological robustness of the Phylogenetic tree. Isolates from this study are presented in red. *Bacillus stercoris* strain HY38 was used as an outgroup to root the tree.

Isolates CP3 and AL3 formed a clade with *Staphylococcus saprophyticus* of accession number PP390036.1. Within the clade there were also strains of *S. pseudoxylosus* and *S. xylosus* (Figure 2).

### 3.3 In Vitro Growth Inhibition of *P. infestans*, *C. siamense*, and *C. sublineola*

The 14 endophytic bacteria identified from the preliminary antagonistic screening against *C. siamense* also exhibited inhibitory properties on growth of other fungal pathogens *P. infestans* and *C. sublineola* in dual culture assays. The growth inhibition by the endophytic bacteria ranged from 23.3%−72.3% for *C. siamense*, 27.7%−66.8% for *C. sublineola* and 24.3%−75.3% for *P. infestans* with significant differences among the endophytic bacteria (*p* < 0.05) (Table 3).

**Table 3:** *In vitro* antifungal activity of the 14 potential endophytic bacteria isolates against three fungal pathogens under *in vitro* dual culture after 10 days of incubation.

ID of endophytic bacteria isolate	Fungal pathogen growth inhibition percentages (%)		
	*P. infestans*	*C. siamense*	*C. sublineola*
AL3	47.67 ± 1.3^efgh^	50.63 ± 2.8^cd^	44.32 ± 3.6^cdef^
AS3	75.3 ± 6.4^a^	70.6 ± 3.47^a^	66.8 ± 0.6^a^
BL3	51.13 ± 3.5^defg^	37.36 ± 1.6^cd^	39.78 ± 1.93^bcde^
BP2	54.12 ± 0.29^cdef^	53.62 ± 1.78^c^	46.24 ± 3.7^bc^
CL1	54.13 ± 2.23^cdef^	54.82 ± 1.09^c^	48.17 ± 3.79^bcd^
CP3	51.1 ± 3.95^defg^	50 ± 1.79^cd^	42.34 ± 1.63^bcde^
CS3b	71.1 ± 3.03^ab^	72.3 ± 6.24^a^	65.1 ± 5.43^a^
CS5	44.06 ± 3.65^gh^	36.13 ± 1.21^f^	34.65 ± 1.33^fg^
DL3	45.86 ± 2.24^fgh^	51.21 ± 1.05 d^e^	44.89 ± 0.97^defg^
DL4	38.84 ± 1.06^h^	36.15 ± 0.76^f^	34.03 ± 3.67^g^
DL6	64 ± 1.56^bc^	62.0 ± 5.59^b^	63.0 ± 2.22^a^
DP1	70.3 ± 6.4^ab^	71.5 ± 1.92^a^	66.6 ± 2.04^a^
DP3	58.8 ± 1.34^cd^	56.63 ± 1.12^bc^	50.67 ± 1.15^b^
DP6	57.58 ± 4.43^cde^	53.02 ± 1.07^bc^	47.45 ± 1.0^bc^

*Note:* Data presented in the table is percentages of fungal growth inhibition by endophytic bacteria isolates. Data represents mean ± standard deviation for three replicates. Different lowercase letters on the same column show significant differences in the inhibition percentages (*p* < 0.05) using Tukey's HSD test and ANOVA.

**Figure 3:**
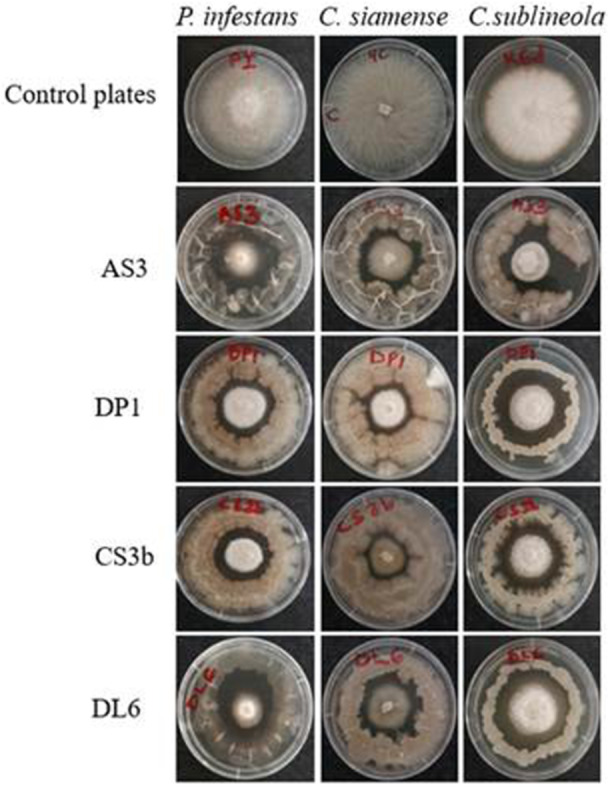
*In vitro* antagonistic activity of *Bacillus siamensis* AS3, *B. velezensis* DP1, *B. velezensis* CS3b, and *B. subtilis* DL6 against the three phytopathogenic fungi based on dual culture plate assay. Figures show growth of bacterial and fungal isolates on PDA media plates. First row from left to right shows the control plates of fungal isolates. Subsequent rows from left to right show treatment plates with fungal isolates grown centrally in the above order with bacterial isolates growing around them in a circular pattern. All treatment plates show zones of inhibition of mycelia growth by the endophytic bacteria isolates.

Four endophytic bacteria isolates namely *B. siamensis* AS3, *B. velezensis* DP1, *B. velezensis* CS3b and *B. subtilis* DL6 (Figure 3) with growth inhibitions above 60% for all the three fungal pathogens were selected for the subsequent experiments. *Bacillus siamensis* AS3 had the highest inhibition of growth of pathogens *P. infestans* (75.3%) and *C. sublineola* (66.8%) while *B. velezensis* CS3b had the highest antagonistic effect on growth of *C. siamense* (72.3%). There was no statistically significant differences among the four endophytic bacteria isolates in the inhibition of mycelia growth for *P. infestans* (*p* = 0.271), *C. siamense* (*p* = 0.0864) and *C. sublineola* (*p* = 0.346).

### 3.4 Antifungal Activity of Selected Endophytic Bacteria VOCs Against *P. infestans*, *C. siamense*, and *C. sublineola*

**Figure 4:**
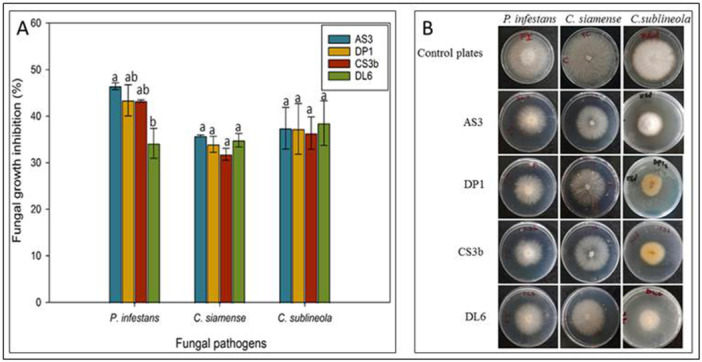
(A) Effect of remote confrontation (bacterial volatile organic compounds) on fungal growth rate. Error bars represent means ± standard errors of replicates. Values with similar lower‐case letters on the bar plots indicate no statistically significant differences at *p* ≤ 0.05 for each grouped data. (B) Growth of fungal isolates on PDA media plates in the presence of bacterial VOCs in a microcosm setup. First row from left to right shows the control plates of fungal isolates while subsequent rows show treatment plates.

The four potential endophytic bacteria isolates (*B. siamensis* AS3, *B. velezensis* DP1, *B. velezensis* CS3b and *B. subtilis* DL6) were tested for antagonistic activities against fungal mycelia growth by production of volatile organic compounds. All the selected endophytic bacterial isolates produced VOCs that suppressed the mycelial growth of the fungal pathogens with varied levels of antagonism (Figure 4). *Phytophthora infestans* was significantly (*p* < 0.05) inhibited by 46.4% (*B. siamensis* AS3), 43.4% (*B. velezensis* DP1), 43.3% (*B. velezensis* CS3b) and 34.1% (*B. subtilis* DL6) at 10 days post incubation. In *C. siamense*, *B. siamensis* AS3 had the highest inhibition (35.8%) while *B. velezensis* CS3b had the least inhibition (31.9%). Isolate *B. subtilis* DL6 had the highest suppression effect (39.6%) on growth of *C. sublineola* while *B. velezensis* CS3b had the least effect (33.9%) (Figure 4). There was no significant difference (*p* > 0.05) among the four bacterial endophytes on growth inhibition of the fungal pathogens *C. siamense* and *C. sublineola* while for *P. infestans* growth a significant difference (*p* < 0.05) was observed (Figure 4).

### 3.5 Effect of Cell‐Free Supernatants (Agar‐Diffusible Metabolites) Against *P. infestans*, *C. siamense* and *C. sublineola*

**Figure 5:**
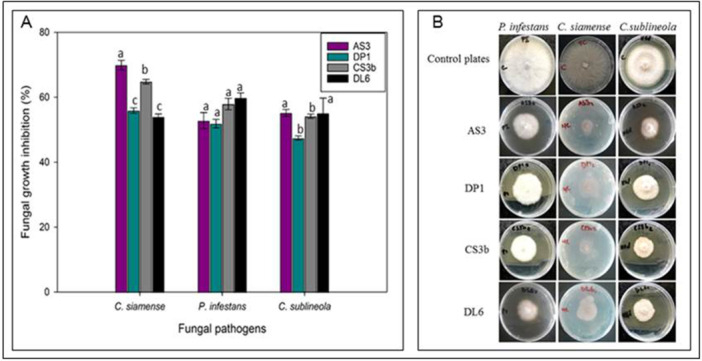
(A) Effect of indirect confrontation (cell free supernatants) on fungal growth rate. Error bars represent means ± standard errors of replicates. Values with similar lower‐case letters on the bar plots indicate no statistically significant differences at *p* ≤ 0.05 for each grouped data. (B) Growth of fungal isolates on PDA media containing endophytic bacteria. First row from left to right shows the control plates of fungal isolates while subsequent rows show treatment plates.

Cell‐free supernatant (CFS) from the selected four endophytic bacteria isolates suppressed/inhibited mycelial growth of the three fungal pathogens (Figure 5). Cell‐free supernatants from isolate *B. subtilis* DL6 demonstrated the highest inhibitory effect on *P. infestans* growth (59.8%) while that from isolate *B. velezensis* DP1 recorded the lowest (51.9%). For *C. siamense*, CFS from *B. siamensis* AS3 showed the highest inhibition (69.9%) of mycelial growth while *B. subtilis* DL6 recorded the lowest (54%). CFS from *B. siamensis* AS3 achieved the highest inhibition of mycelial growth of *C. sublineola* (55.2%) while *B. velezensis* DP1 recorded the lowest inhibition (48%). There was a significant difference (*p* < 0.05) in mycelial growth inhibition by CFS from the four endophytic bacteria in the case of *C. siamense* and *C. sublineola*. There was no significant difference (*p* > 0.05) in mycelial growth inhibition of *P. infestans* by CFS from the four endophytic bacteria (Figure 5).

### 3.6 Identification of Endophytic Bacteria VOCs Using GC‐MS Analysis

**Figure 6:**
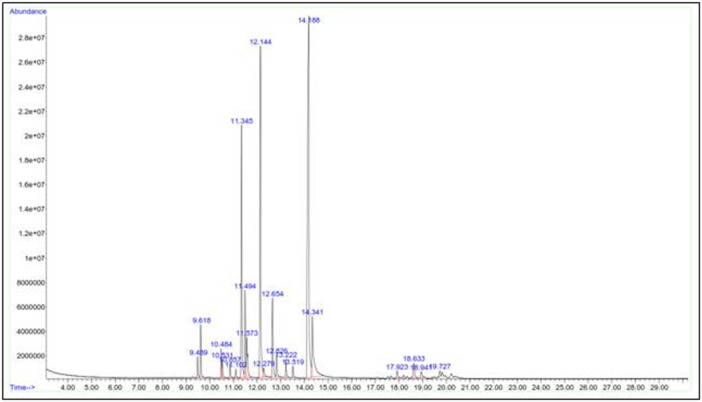
Gas chromatography‐mass spectrometry spectrograms showing peaks of volatile organic compounds produced by endophytic bacteria *Bacillus siamensis* AS3. Peaks are annotated with their percentage abundance.

The endophytic bacteria isolate (*B. siamensis* AS3) with the highest antifungal activity against *P. infestans*, *C. siamense* and *C. sublineola* was selected for GC‐MS analysis. GS‐MS analysis revealed the presence of volatile compounds produced by *B. siamensis* AS3 isolate (Figure 6). A total of 20 volatile compounds were detected based on GC‐MS analysis, of which 13 compounds have been reported to exhibit antifungal properties (Supporting Information S1: Table S2). Compounds tridecylamine, O‐xylene, cetane, butyl benzene and 2,3,5‐trimethylhexane with percentage abundance of 14.64%, 13.03%, 12.76%, 12.5% and 11.02%, respectively, were produced by *B. siamensis* AS3. The othr compounds exhibited lower abundance ranging from 0.33% to 10.15%.

### 3.7 Physiological Characterization of the Endophytic Bacteria

**Figure 7:**
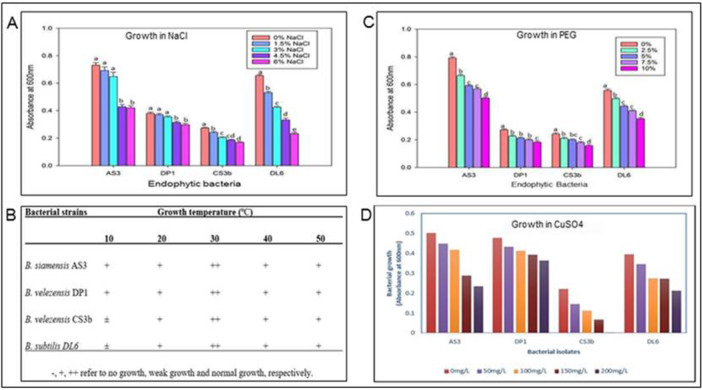
Growth of endophytic bacteria in (A) 0%−6% NaCl concentrations, (B) 10°C−50°C temperatures, (C) 0%–10% PEG concentrations, and (D) 0−200 mg L^−^^1^ CuSO_4_ concentrations. Error bars represent means ± standard errors of replicates. Values with similar lower‐case letters on the bar plots indicate no statistically significant differences at *p* ≤ 0.05 for each grouped data (for each endophytic bacteria).

All the four potential endophytic bacteria isolates tested for their salt tolerance were able to maintain growth in 0%−6% NaCl (Figure 7). Growth rate however decreased as the salt concentration increased with a significant difference in growth (*p* < 0.05). The bacteria isolates also exhibited tolerance to osmotic stress by growth in media containing up to 10% osmotic stress‐inducing polyethylene glycol (PEG). There was a significant reduction in growth (*p* < 0.05) with increase in PEG concentration for all the four endophytic bacteria. All the isolates exhibited growth capabilities across temperatures ranging from 10°C−50°C. The optimal growth was observed at a temperature of 30°C with reduction in growth to the higher and lower temperatures. The endophytic bacteria isolates exhibited growth in the presence of metal ions by growth in CuSO_4_ up to 200 mg L^−^^1^ except isolate *B. velezensis* CS3b which only tolerated growth up to 150 mg L^−1^ (Figure 7).

### 3.8 Plant Growth Promotion Activity of Selected Endophytic Bacteria

Significantly higher growth parameters were observed in plants inoculated with endophytic bacteria than in non‐inoculated control plants (*p* < 0.05). The heights of cassava plants due to endophytic bacteria inoculations were significantly increased (*p* < 0.05) compared to the non‐inoculated plants. The growth performance, including the fresh and dry weights for whole plants, leaf number, root length and numbers differed significantly (*p* < 0.05) compared with the non‐inoculated plants (Table 4).

**Table 4:** Effect of selected antagonistic endophytic bacteria isolates on growth promotion potential in inoculated plants cassava cultivar KALRO Agric under greenhouse conditions.

Bacterial isolate	Plant height (cm)	Root length (cm)	No. of leaves	No. of roots	Fresh weight (g)	Dry weight (g)
Control	7.4 ± 3.05^b^	8.0 ± 0.1^b^	7.0 ± 0.7^b^	7.0 ± 0.7^b^	4.6 ± 0.3^b^	1.2 ± 0.2^b^
AS3	11.7 ± 1.6^a^	14.1 ± 0.9^a^	9.3 ± 0.4^a^	15.3 ± 1.1^a^	6.1 ± 0.4^a^	2.8 ± 0.2^a^
DP1	12.4 ± 0.9^a^	11.1 ± 1.8^ab^	9.7 ± 0.4^a^	16.3 ± 1.1^a^	7.2 ± 0.8^ab^	3.3 ± 0.4^a^
CS3b	12.2 ± 1.2^a^	10.5 ± 0.5^ab^	9.3 ± 0.4^a^	10.7 ± 0.4^a^	5.5 ± 0.2^ab^	2.8 ± 0.1^a^
DL6	11.6 ± 0.6^a^	13.1 ± 2.4^ab^	10.0 ± 0.7^a^	10.0 ± 0.7^b^	6.4 ± 0.9^ab^	2.9 ± 0.2^a^

*Note:* Data represents means ± standard error of biological replicates. Values within the same column with different lowercase letters are significantly different from each other at (*p* < 0.05) using Tukey's HSD test and ANOVA. Control is non‐inoculated plants.

## 4 Discussion

The current study explored the potential of endophytic bacteria isolated from leaves, stems and petioles of four cassava cultivars for the biocontrol of fungal diseases infecting cassava, sorghum and potato as well as the ability to promote growth in cassava. In the current study, 84 endophytic bacteria isolates were obtained from cassava plants to determine their antagonistic potential against *P. infestans*, *C. siamense* and *C. sublineola* infecting potato, cassava and sorghum. Initial screening confirmed antagonistic potential of 14 endophytic bacteria isolates against the fungal pathogen *C. siamense* in comparison to the control. Using the ribosomal region of the 16S rRNA, 12 and 2 endophytic bacteria isolates were confirmed to belong to the genus *Bacillus* and *Staphylococcus*, respectively. The predominance of the genus *Bacillus* as bacterial endophytes has also been reported from several healthy perennial and annual plants (AlAli et al. 2022). *Bacillus* species are known to ubiquitously inhabit a wide range of ecological niches and form part of the endophytic communities in many plants. This study revealed 14 endophytic bacteria isolates with potential biocontrol activity against *P. infestans*, *C. siamense*, and *C. sublineola* pathogens, corresponding to *Bacillus siamensis* (AS3), *B. velezensis* (DP1 and CS3b), *B. subtilis* (DL6), *B. cereus* (DL3, DP3 and DL4), *B. safensis* (CL1 and CS5), *B. aerophilus* (BL3), *B. altitudinus* (BP2) and *Staphylococcus saprophyticus* (CP3 and AL3). *Bacillus siamensis*, *B. velezensis*, *B. safensis*, *B. subtilis*, *B. cereus* and *B. aerophilus* are well known biocontrol agents that suppress phytopathogenic fungi from various plants (Sharma et al. 2021). For instance, *B. siamensis* isolated from various crops have been shown to have inhibitory properties on growth of various fungal pathogens of crops such as potato, rice and chickpea (Sharma et al. 2021; Gorai et al. 2021; Yang et al. 2023).

Selected isolates of *B. velezensis* DP1 and CS3b, *B. siamensis* AS3 and *B. subtilis* DL6 showed the highest antifungal effect when challenged directly against the pathogens and indirectly through the use of their volatile organic compounds and agar‐diffusible metabolites *in vitro*. The selected endophytic bacteria demonstrated inhibition levels of more than 60% against three fungal pathogens *Colletotrichum siamense*, *C. sublineola*, and *P. infestans*. The clear zone of inhibition without physical contact between the endophytic bacterial and fungal pathogens may be due the fact that these endophytic bacteria produce active antifungal substances that impede fungal mycelia growth (Sharma et al. 2021). The bacterial endophytes could also compete with the pathogens for nutrients and thereby suppress fungal mycelial growth (Baard et al. 2023).

The effect of VOCs produced by selected endophytic bacteria isolates on the mycelial growth of *P. infestans*, *C. siamense*, and *C. sublineola* was also studied in order to elucidate other potential mechanisms of inhibition. VOCs have many functions as signalling molecules and, among them, they can have antifungal properties against different plant pathogens (Bustamante et al. 2022). Endophytic bacteria *B. velezensis* DP1 and CS3b, *B. siamensis* AS3 and *B. subtilis* DL6 produced VOCs which inhibited the fungal mycelia growth of the three phytopathogenic fungi. Volatile organic compounds produced by microbial communities participate in species communication and are widely studied for their antifungal and antibiotic properties (Josselin et al. 2022). VOCs derived from endophytes aid in disease control by acting as antimicrobial agents and inducing pathogen resistance in plants hence preventing pathogen colonization (Tilocca et al. 2020). Sdiri et al. (2022) used olive endophytes in the biocontrol of olive anthracnose pathogen *Colletotrichum acutatum* reducing disease severity and incidence. The VOCs produced by the endophytic bacteria *B. velezensis* DP1 and CS3b, *B. siamensis* AS3 and *B. subtilis* DL6 significantly inhibited the growth of the three phytopathogenic fungi. However, the antifungal efficacy of VOCs was significantly lower than using the living bacterial cells in the *in vitro* dual culture assays. This is due to the synergistic effects of antifungal compounds released by bacterial cells in addition to the VOCs in the dual culture assays. Boiu‐Sicuia et al. (2023) reported similar findings with significant differences in inhibitory activities of *Bacillus* species in dual culture and VOC inhibition assays.

CFSs from endophytic bacteria showed inhibitory effects on growth of the three fungal pathogens. CFS is liquid derived from microbial growth and contains metabolites resulting from the growth along with residual nutrients from growth media (Mani‐López et al. 2022). CFS from endophytic bacteria *B. velezensis* DP1 and CS3b, *B. siamensis* AS3 and *B. subtilis* DL6 significantly inhibited growth of the three fungal pathogens *C. siamense, C. sublineola* and *P. infestans*. *Bacillus* species secrete diverse range of secondary metabolites that are highly inhibitory against plant pathogens (Zhao et al. 2022). For example, *B. velezensis* secretes antibiotics such as bacillopeptines, macrolactins, bacillaene, difficidin, amylolysin, bacilysin, lantipeptides and microcins, cell‐wall degrading enzymes such as chitinase, protease and β−1,3‐glucanase, antimicrobial polypeptides such as iturins, fengycins, and surfactins and siderophores such as bacillibactin (Choub et al. 2021; Liu et al. 2019; Pan et al. 2017; Chen et al. 2007). This can explain the inhibitory effect on growth of the fungal pathogens in this study. CFS from *B. subtilis* isolates inhibited the growth of root pathogen *Fusarium verticillioides* (Hirozawa et al. 2024) while that of *B. velezensis* isolate inhibited growth of *Botrytis cinerea* (Zhao et al. 2022). The current study therefore supports previous studies on the role of bacterial CFS in biocontrol of fungal pathogens. This study supports previous reports that *Bacillus* species produce different secondary metabolites and antagonistic substances such as antibiotics. The findings on the antagonistic activities of cassava endophytic bacteria in various assays against fungal pathogens of three crops shows their potential in broad‐spectrum biocontrol of fungal pathogens in other crops.

Bacterial endophytes are a reliable source of bioactive compounds that can be used to control plant diseases (Sharifi and Ryu 2016). In our study, GC‐MS was used to detect volatile bioactive antifungal compounds in the extracts of the most potent antagonistic endophytic bacteria *B. siamensis* AS3. From the GC‐MS analysis, *B. siamensis* AS3 was found to produce 20 VOCs, of which 12 have been reported to have known antifungal properties. These bioactive compounds showed large peaks in the cell‐free extracts of the endophytic bacteria *B. siamensis* AS3, indicating that they contribute significantly to the antifungal activities. It was found that alkanes (cetane, 2,3,5‐Trimethylhexane) and benzene derivatives (N‐propyl benzene, butyl benzene) had the greatest peaks of all the volatile secondary metabolites in substrate of the endophytic bacteria *B. siamensis* AS3. This confirms previous reports that these compounds are responsible for a wide range of antifungal activities in biocontrol potential including induction of systemic resistance in plants, modulation of pathogen gene expression and pathogen cell alterations (Ajuna et al. 2024; Grahovac et al. 2023; Card et al. 2016). *Bacillus siamensis* from blueberry has been reported to produce M‐xylene, 1‐heptanol, and 2‐ethylhexanol, which are effective against post‐harvest grey mold pathogen *Botrytis cinerea*. Álvarez‐García et al. (2020) and Yuan et al. (2012) reported the volatile compounds cetane and N‐propyl benzene, respectively, from *B. siamensis* to possess antifungal properties against *Fusarium oxysporum*. Additionally, *B. siamensis* has been found to secrete many antifungal metabolites which can inhibit several fungal pathogens (Alijani et al. 2019).

The abiotic stress tolerance including salinity, drought stress, temperature and copper ions of the antagonistic endophytic bacteria *B. velezensis* DP1, *B. velezensis* CS3b, *B. siamensis* AS3 and *B. subtilis* DL6 was tested *in vitro*. The antagonistic endophytic bacteria were able to grow in 6% NaCl, 10% PEG, and at temperatures up to 50°C. This characteristic feature makes these endophytic bacteria potential ideal candidate that can be harnessed in saline‐prone regions to enhance agricultural productivity. The findings on thermotolerance suggests that the four antagonistic endophytic bacteria may be simple/moderate thermophiles, as they are able to grow under heat stress conditions up to 50°C. Copper fungicides are frequently used in the chemical control of plant fungal pathogens (Ferreira et al. 2014). The ability of endophytic bacteria particularly *B. siamensis* AS3 and *B. subtilis* DL6 to survive in high copper ion concentrations of 200 mg L^−^^1^ of CuSO_4_ indicate their potential use in soils where these fungicides have been used. The adaptability of the four antagonistic endophytic bacteria to variations in salinity, temperature and drought as well as copper toxicity make them suitable biological control agents (BCAs) candidates for a broad range of ecosystems and can allow their use to reach the desired beneficial effects. However, it is of importance to establish whether these bacteria can also interact beneficially with crop plants in greenhouse experiments and finally open agriculture.

Plant growth‐promoting activities of four antagonistic endophytic bacteria *B. velezensis* DP1, *B. velezensis* CS3b, *B. siamensis* AS3 and *B. subtilis* DL6 was tested on cassava seedling plants. The inoculated cassava plants showed better growth compared to that of the non‐inoculated control plants. Growth parameters were significantly improved in inoculated plants compared to non‐inoculated plants. Similar results were produced when endophytic bacteria *B. velezensis*, *B. siamensis*, *B. subtilis*, and *B. siamensis* were applied to plants of wheat, chickpea, pepper, tomato and rice (Shen et al. 2023; Huang et al. 2023; Peterson et al. 2022; Gorai et al. 2021; Miljaković et al. 2020; Dahiya et al. 2019). These studies reported that the improved plant growth upon inoculation with the bacteria endophytes might be due to the plant growth‐promoting potential of the inoculated bacteria. Similarly, in the present study, the growth improvement of inoculated cassava plants might be due to the biochemical and growth promoting effects of the endophytic bacteria *B. velezensis* DP1, *B. velezensis* CS3b, *B. siamensis* AS3 and *B. subtilis* DL6. Further studies on plant growth promotion mechanisms of these endophytic bacteria *in vitro* can give insights on the effect of their inoculation on cassava plants.

The use of strains of *Bacillus* species and their derived products are widely marketed and utilized in modern agricultural systems and have achieved significant benefits worldwide for the biocontrol of plant diseases. As microbial biological agents, different strains of *Bacillus* species including *B. velezensis* DP1, *B. velezensis*, *B. siamensis* and *B. subtilis* have been formulated as commercial biopesticides for suppression of various plant phytopathogens and growth promotion (Fatima et al. 2023; Zhang et al. 2023). However, commercial biopesticides derived from *Bacillus* species have not completely replaced the use of chemical fungicides due their limited efficacy in natural conditions. The practical utilization of biological control agents from Bacillus species is confronted with unstable disease suppression efficacy under field conditions, which limits the applicability of the biocontrol products (Ling et al. 2010). The colonization and functional efficacy of inoculated *Bacillus* biological agents is complicated by the dynamic and complex soil‐plant‐microbe interactions, effects of climate change and is also affected by many factors such as soil characteristics, indigenous microbiota and plant species and/or genotype (Xiong et al. 2017). There is need to explore the new approaches proposed such as the use of antagonistic metabolites instead of biocontrol agents to improve the biocontrol efficiency.

The findings of the current study indicated that *B. velezensis*, *B. siamensis* and *B. subtilis* selected through *in vitro* antagonistic experiments exhibited potential of broad‐spectrum biological control against three crop phytopathogenic fungi. However, the limitation of the current study was that biocontrol assay of *B. velezensis*, *B. siamensis* and *B. subtilis* against the three crop pathogenic fungi was not performed on plants. Therefore *in vivo* assessments could be necessary to validate the antifungal efficacy of *Bacillus* species against *C*. *siamense*, *C*. *sublineola*, and *P. infestans*.

## 5 Conclusions

The findings from this study revealed that the leaves, stems and petioles of cassava contain a robust source of potential biological control agents against *P. infestans*, *C. siamense* and *C. sublineola*. Our selected endophytic bacteria isolates, especially the ones identified as *B. velezensis* DP1, *B. velezensis* CS3b, *B. siamensis* AS3 and *B. subtilis* DL6 demonstrated broad‐spectrum antagonistic activity against three phytopathogenic fungi based on dual culture, indirect and remote confrontation assays. Different tests also confirmed that the selected four endophytic antagonistic bacteria had good plant growth‐promoting properties and plant abiotic stress tolerance abilities. The four identified *Bacillus* species have a potential to be utilized for the production of bio‐pesticides for management of fungal diseases and in the production of biofertilizers. These findings hold potential for environmentally friendly strategies to manage anthracnose and late blight diseases in cassava, sorghum and potato crops. There is need to evaluate the selected isolates of *B. velezensis*, *B. siamensis* and *B. subtilis* on field trials for their prevention and curative abilities against *P. infestans*, *C. siamense* and *C. sublineola*.

## Author Contributions

**Roselyne Nyawir Owino:** conceptualization, methodology, investigation, data curation, data analysis, validation, writing – original draft, writing – review and editing. **Edward K. Nguu:** conceptualization, methodology, investigation, validation, supervision, writing – review and editing. **George O. Obiero:** conceptualization, methodology, investigation, writing – review and editing. **Evans N. Nyaboga:** conceptualization, methodology, investigation, validation, data analysis, writing – review and editing. All the authors have read and agreed to the published version of the manuscript.

## Funding

The authors received no specific funding for this work.

## Ethics Statement

The authors have nothing to report.

## Conflicts of Interest

The authors declare no conflicts of interest.

## Supporting information

Supplementary File.

## Data Availability

The data that supports the finding of this study are included in this article and its supplementary material. Further inquiries on raw data can be availed by the corresponding author upon request.
