# Supplementary material for: Cassava Endophytic Bacteriome as Potential Biocontrol Agents Against Three Crop Phytopathogenic Fungi
Source: Microbiologyopen. 2026 Feb 23;15(2):e70254. doi: 10.1002/mbo3.70254 (PMC12929919; doi:10.1002/mbo3.70254)
Supplement: Supplementary file 1 — Supplementary File. [file MBO3-15-e70254-s001.docx]

**Cassava Endophytic Bacteriome as Potential Biocontrol Agents against Three Crop Phytopathogenic Fungi**

Roselyne Nyawir Owino^1,2^, Edward K. Nguu^1,2^, George O. Obiero^1,2^ and Evans N. Nyaboga^1,2^*

^1^Department of Biochemistry, University of Nairobi, P.O. Box 30197 - 00100, Nairobi, Kenya

^2^Centre for Biotechnology and Bioinformatics, University of Nairobi, P.O. Box 30197 - 00100, Nairobi, Kenya

*Correspondence: nyaboga@uonbi.ac.ke

**Supplementary Tables**

**Supplementary Table S1**: Endophytic bacteria isolated from different plant tissues of the 4 cassava cultivars

| **Cassava cultivar** | **Endophytic bacteria from different plant tissues** | | | | | | |
| --- | --- | --- | --- | --- | --- | --- | --- |
|  | **Leaf** | | **Stem** | | **Petiole** | | **Total No. of isolates** |
|  | **No. of isolates** | **ID of endophytic bacterial isolates** | **No. of isolates** | **ID of endophytic bacterial isolates** | **No. of isolates** | **ID of endophytic bacterial isolates** |  |
| 1. MM08/2206 | 6 | AL1, AL2, AL3, AL4, AL5, AL6 | 6 | AS1, AS2, AS3, AS4, AS5, AS6 | 5 | AP1, AP2, AP3, AP4, AP5 | 17 |
| 1. SAMGOJA | 7 | BL1, BL2, BL3, BL4, BL5, BL6, BL7 | 6 | BS1, BS2, BS3, BS4, BS5, BS6 | 7 | BP1, BP2, BP3, BP4, BP5, BP6, BP7 | 20 |
| 1. KAREMBO | 9 | CL1, CL2, CL3, CL4, CL5, CL6, CL7, CL8, CL9 | 7 | CS1, CS2, CS3, CS3b, CS4, CS5, CS6 | 6 | CP1, CP2, CP3, CP4, CP5, CP6 | 22 |
| 1. MM96/4884 | 8 | DL1, DL2, DL3, DL4, DL5, DL6, DL7, DL8 | 7 | DS1, DS2, DS3, DS4, DS5, DS6, DS7 | 10 | DP1, DP2, DP3, DP4, DP5, DP6, DP6b, DP7, DP8, DP9 | 25 |

The ID of the endophytic bacterial isolate names were given according to the cassava cultivars (first letter) and tissues (second letter) used for isolation of endophytic bacteria followed by the serial number. The cultivar names are represented by letters A, B, C and D and the tissues used for isolation of endophytic bacteria are represented by: L - leaf, P - petiole and S - stem.

**Supplementary Table S2:** Volatile compounds from gas chromatography-mass spectrometry (GC-MS) analysis of endophytic bacteria *B. siamensis* (isolate AS3)

| **Retention Time (min)** | **Constituent** | **% abundance** | **Chemical formula** | **Nature of compound** | **Molecular weight (g/mol)** | **Reported antifungal activity** |
| --- | --- | --- | --- | --- | --- | --- |
| 9.459 | 2-propanamine | 7.860 | C3H9N | Amine | 59.11 | Antagonist of *Botrytis cinerea* (Sánchez-Hernández *et al*., 2024) |
| 10.484 | P-Xylene | 0.222 | C_6_H_4_(CH_3_)_2_ | Xylene | 106.16 | Antifungal effect on oat leaf strife pathogen *Pyrenophora avenae* (Brear *et al*., 1997) |
| 10.531 | 2,4 –Dimethyl hexane | 0.913 | C8H18 | Alkane | 114.23 | No significant reports on antifungal activity |
| 10.587 | 2,3,5 -Trimethylhexane | 11.022 | C9H20 | Alkane | 128.25 | No reported antifungal activity |
| 11.103 | Ethyl benzene | 5.000 | C8H10 | Aromatic hydrocarbon/ benzene derivative | 7500 | Antagonistic effect on *Fusarium oxysporum* f. sp*. cubense* (Yuan *et al*., 2012) |
| 11.345 | 1,2 -Dimethylbenzene/ O-Xylene | 13.034 | C_6_H_4_(CH_3_)_2_ | Cyclic hydrocarbon/ xylene | 106.16 | Antagonistic on strawberry anthracnose pathogen *Colletotrichum nymphaeae* (Alijani *et al*., 2019) |
| 11.404 | Tridecane | 0.555 | C13H28 | Alkane | 184.36 | Antagonistic on *Alternaria alternata* (Gil *et al*., 2023); growth promotion in olives (Montes-Osuna *et al*., 2022) |
| 11.573 | 1,3 -Dimethylbenzene/ M-Xylene | 1.708 | C_6_H_4_(CH_3_)_2_ | Cyclic hydrocarbon/ xylene | 106.16 | Growth inhibition of post-harvest grey mold fungus *Botrytis cinerea* (Wang *et al*., 2022) |
| 12.144 | N –Propyl benzene | 10.148 | C9H12 | Aromatic hydrocarbon/ benzene derivative | 120.19 | Antagonistic effect on *Fusarium oxysporum* f. sp*. cubense* (Yuan *et al*., 2012) |
| 12.279 | Benzene butyl/ Butyl benzene | 12.496 | C10H14 | Aromatic hydrocarbon/ benzene derivative | 134.22 | No reported antifungal activity |
| 12.654 | Phthalan | 0.268 | C8H8O | Benzofurans | 120.15 | Moderate antifungal properties (Karmakar *et al*., 2009) |
| 12.826 | 2,2 –dimethylhexane | 0.847 | C8H18 | Alkane | 114.23 | No significant reports on antifungal activity |
| 13.222 | 2-oxopropanoic acid/ pyruvic acid | 4.123 | C3H4O3 | Pyruvate/ carboxylic acid | 88.06 | Enhancement of antifungal properties of lactic acid bacteria (Valerio *et al*., 2016) |
| 13.519 | Dihexylether | 1.458 | C12H26O | Ether | 186.33 | No significant reports |
| 14.188 | Tridecylamine | 14.637 | C13H29N | Amine | 199.38 | No significant reports |
| 14.341 | Cetane/ Hexadecane | 12.756 | C16H34 | Alkane | 226.44 | Antifungal properties on *Fusarium oxysporum* (Alvarez-Garcia. *et al*., 2020) |
| 17.923 | Acetic acid butyl ester/ Butyl acetate | 0.304 | C6H12O2 | Acetate | 116.16 | Growth inhibitor of oil palm basal stem rot pathogen Ganoderma boninense (Rupaedah *et al*., 2024) |
| 18.633 | Isooctanol/ 6-Methyl-1-heptanol | 1.036 | C8H18O | Alcohol | 130.23 | Biocidal effects on *Fusarium oxysporum* (Ye *et al*., 2020) |
| 18.919 | Phenylethylalcohol | 0.275 | C_6_H_5_CH_2_CH_2_OH | Alcohol | 122.16 | Antifungal agent of *Candida sp* (Majdabadi *et al*., 2018) |
| 19.277 | 2,2-dimethoxy-1,2-diphenyl- Ethanone | 0.388 | C16H16O3 | Ketone | 256.3 | No reported antifungal activity |

**Supplementary Figures**


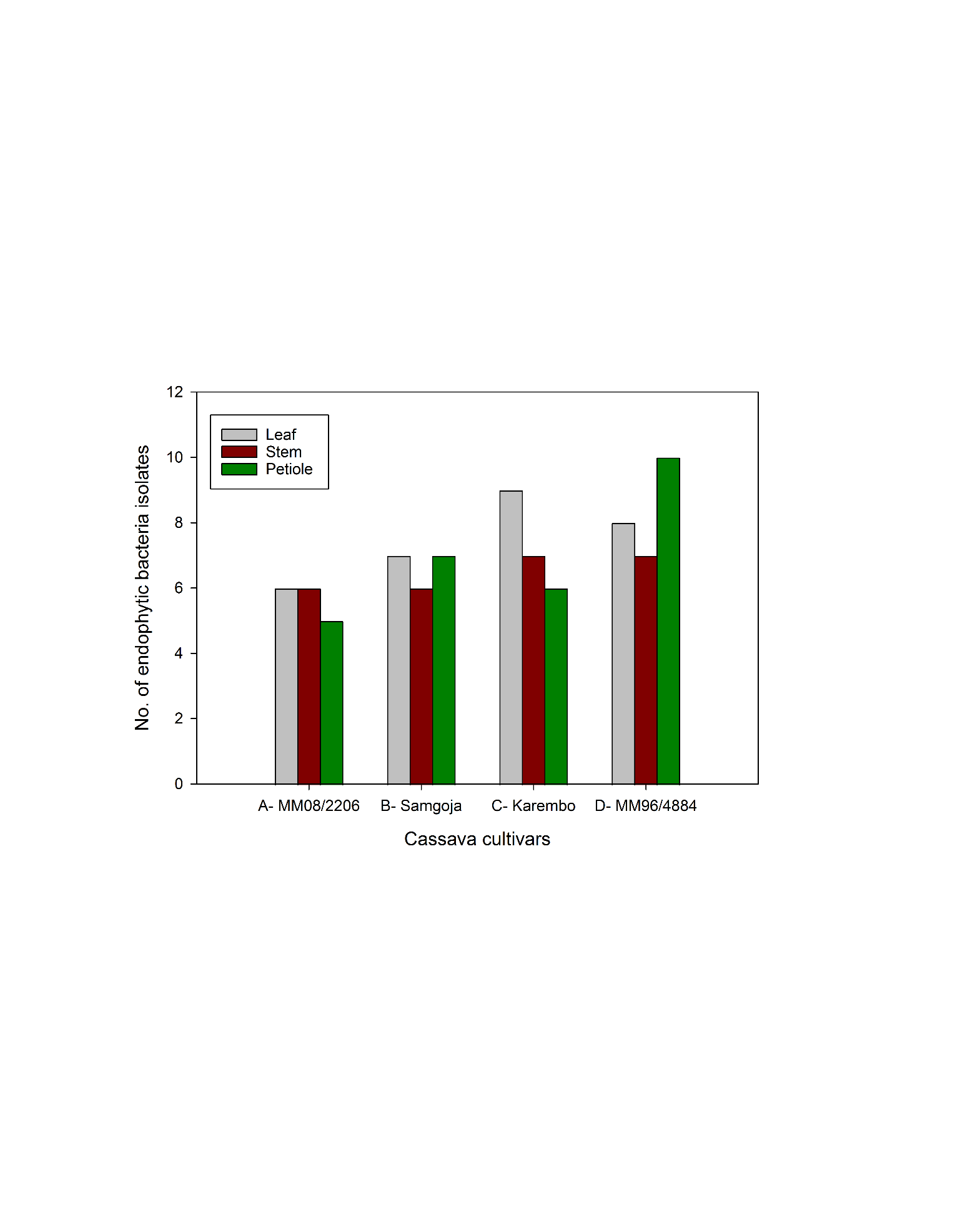


**Supplementary Figure S1**: The number of endophytic bacteria isolated from leaf, stem and petiole tissues of four cassava cultivars used in the study.
